# Transcriptome-wide analysis associates *ID2* expression with combined pre- and post-capillary pulmonary hypertension

**DOI:** 10.1038/s41598-019-55700-y

**Published:** 2019-12-20

**Authors:** Meghan J. Arwood, Nasim Vahabi, Christelle Lteif, Ravindra K. Sharma, Roberto F. Machado, Julio D. Duarte

**Affiliations:** 1 Department of Pharmacotherapy and Translational Research, College of Pharmacy, University of Florida, Gainesville, FL USA; 2 Department of Physiology and Functional Genomics, College of Medicine, University of Florida, Gainesville, FL USA; 3 Department of Medicine, Division of Pulmonary, Critical Care, Sleep, and Occupational Medicine, Indiana University, Indianapolis, IN USA

**Keywords:** Genetics research, Translational research

## Abstract

Heart failure with preserved ejection fraction (HFpEF) patients who develop pulmonary hypertension (PH) have an increased risk of death, with combined pre- and post-capillary PH (CpcPH) having the highest risk. However, the mechanism behind PH development in HFpEF is poorly understood. We aimed to identify transcriptomic associations with PH development in HFpEF. Blood was collected from 30 HFpEF patients: 10 without PH, 10 with isolated post-capillary PH, and 10 with CpcPH. Gene expression measurements were completed using transcriptome-wide RNA sequencing. Gene expression differences were compared using a quasi-likelihood method adjusting for age, sex, race, and smoking-status. Biological pathways were compared using global gene expression differences. A replication in 34 additional heart failure patients and a validation in lung tissue from a representative mouse model were completed using quantitative PCR. Six differentially expressed genes were identified when comparing transcriptomics between subjects with CpcPH and those without PH. When tested in additional subjects, only the association with *ID2* replicated. Consistent with clinical findings, *Id2* expression was also upregulated in mice with HFpEF and PH. Pathway analysis identified proliferative and mitochondrial pathways associated with CpcPH. Thus, these patients may possess systemic pathophysiological differences similar to those observed in pulmonary arterial hypertension patients.

## Introduction

Pulmonary hypertension (PH) is a complex condition, with the persistence of elevated mean pulmonary arterial pressure (MPAP) leading to right-sided heart failure and ultimately death^1^. PH due to left heart disease (also termed Group 2 PH by the World Health Organization) is one of the most common types of PH, stemming from left ventricular dysfunction and/or valvular heart disease^2^. In Group 2 PH (historically defined as a MPAP ≥25 mmHg and pulmonary arterial wedge pressure (PAWP) >15 mmHg), elevated left-sided filling pressures lead to passive post-capillary pulmonary venous congestion and may lead to additional remodeling effects^3–6^. PH is common in left heart failure (HF), with researchers estimating that 55 to 80% of HF patients develop PH^7–13^. Importantly, both the presence and severity of PH confer additional morbidity and mortality risk in HF patients^11,12^. In general, the five-year mortality rate for HF-PH patients is about 50%^14^.

HF-PH has, in recent years, been categorized into two main hemodynamic phenotypes based on calculations of diastolic pulmonary gradient (DPG) and/or pulmonary vascular resistance (PVR): isolated post-capillary PH (IpcPH; DPG < 7 mmHg and/or PVR ≤ 3 Wood Units) and combined pre- and post-capillary PH (CpcPH; DPG ≥ 7 mmHg and/or PVR > 3 Wood Units)^2,15,16^. While most HF-PH patients present with IpcPH, CpcPH is observed in up to 20% of HF-PH patients^17^. CpcPH patients likely have intrinsic pulmonary vascular disease and MPAP that is disproportionate to the initial increase of left-sided filling pressures. Right ventricular to pulmonary vascular coupling is poor in these patients, which is thought to be one reason for the more than two-fold increased mortality risk compared to IpcPH patients^17,18^.

Since a high percentage of HF patients develop PH and the mortality rate is alarming, effective treatment strategies for this disease are needed, yet no established treatment guidelines currently exist^19^. The current standard of care is to treat comorbid disorders and concentrate on improving volume status and left ventricular relaxation properties^20^. This is particularly problematic for patients with heart failure with preserved ejection fraction, who have few treatment options available to treat their underlying disease. Thus, it is vital to understand the mechanisms underlying PH development in HFpEF patients in order to personalize their treatment regimens and uncover novel treatment strategies. One way to better understand these mechanisms is to identify biological pathways that are associated with HFpEF-PH development. Although recent studies have identified limited candidate genes for HF-PH pathogenesis^21–23^, there is a paucity of research in this area and a lack of robust gene expression analyses. Therefore, the aim of this study was to conduct a transcriptome-wide association study to identify gene expression signatures associated with IpcPH and CpcPH development in HFpEF patients.

## Methods

### Study population

The discovery and replication cohorts used in this study consisted of HF patients with and without PH recruited from the University of Illinois Hospital and Health Science System (UI Health) heart failure and pulmonary hypertension clinics. All patients provided informed, written consent before participation, and protocols for recruitment were approved by the University of Illinois at Chicago Institutional Review Board in accordance with federal and local regulations. Data available for abstraction included patient demographics, past medical history, medication regimen, as well as echocardiogram and right heart catheterization measurements. Additionally, blood samples were collected at the time of enrollment, and RNA was isolated from peripheral blood mononuclear cells (PBMCs) in a subset of subjects.

HFpEF patients were diagnosed with heart failure, had an ejection fraction >45%, and a history of diastolic dysfunction on echocardiogram. In the discovery cohort, PBMCs were available from 30 HFpEF patients: 10 with HFpEF without PH (MPAP ≤ 20 mmHg or tricuspid regurgitation velocity ≤3 m/s measured on echocardiogram], 10 with IpcPH, and 10 with CpcPH.

### Gene expression with RNA-seq

RNA was isolated from PBMCs that were extracted from peripherally drawn blood. After bead-based rRNA depletion was performed, cDNA was synthesized from RNA, and then amplified to create a sequencing library. Sequencing was performed on the Illumina NextSeq 500 platform (Illumina, San Diego, CA, USA), and paired reads of approximately 75 bases were generated. Sequencing was completed in four separate pools, with assignments to each pool being randomized to minimize potential biases from sequencing pool or RNA extraction batch. FASTQ files from sequencing were aligned to the reference genome (hg38) with STAR^24^. STAR was also used to derive the number of reads mapped to each gene/transcript. Genes with low expression were filtered out and data were normalized using the trimmed mean of M-values (TMM) method^25^.

Gene expression levels were compared in pairwise analyses among the three HFpEF groups: IpcPH vs. no PH, CpcPH vs. no PH, and CpcPH vs. IpcPH. These analyses were performed using a negative binomial generalized linear model with a quasi-likelihood method in edgeR^26^, adjusting for age, sex, race, and smoking status. Genes were considered differentially expressed if the false discovery rate (FDR) adjusted q-value ≤ 0.05. A network analysis was completed to determine co-expressed genes using GeneMANIA^27^. A pathway analysis using gene set enrichment was also performed in GAGE^28^, using Kyoto Encyclopedia of Genes and Genomes (KEGG) pathways^29^. Additional information on RNA-seq procedures can be found in the Online Data Supplement.

### Replication analysis

In an attempt to replicate the differentially expressed genes identified using RNA-seq, PBMC gene expression was compared in subjects that were not included in the RNA-seq discovery cohort but were from the same research protocols used to recruit patients at UI-Health. Thus, similar clinical data as described in the discovery cohort were also collected in the replication cohort. These subjects were categorized into groups of no PH, IpcPH and CpcPH using the criteria described above. However, unlike the discovery cohort above, this replication cohort contained both HFpEF and heart failure with reduced ejection fraction (HFrEF) subjects.

Expression was measured in each of the differentially expressed genes identified in RNA-seq analysis using quantitative real-time PCR (qRT-PCR). RNA was isolated and reverse transcribed to complementary DNA. Gene expression was then measured using TaqMan® gene expression assays (Thermo Fisher Scientific, Waltham, MA, USA). The comparative 2^−ΔCt^ method was used for calculating relative quantitation of gene expression^30^, normalized to the expression of the housekeeping gene *RPLP0*. Pairwise analyses of the no PH, IpcPH and CpcPH groups were completed using a similar regression model as described above, adjusting for identical covariates (age, sex, race, and smoking status). Because our aim was to confirm findings in the discovery cohort, associations with a similar direction of effect and a one-sided *P*-value ≤ 0.05 were considered significant.

### Tissue-specific validation

Male AKR/J mice (The Jackson Laboratory, Bar Harbor, ME) between 3–6 weeks old were randomized (N = 4–5 per group) to receive a high-fat diet (HFD; 60% lipid/kcal; Research Diets, New Brunswick, NJ) or normal chow (control mice) for 19 weeks. This mouse model has been previously shown to consistently develop HFpEF-PH^31^. After 19 weeks, mice were weighed, a blood sample was taken, and hemodynamics were measured via left and right heart catheterization, as previously described^32^. Mice were then euthanized, and a lung tissue sample was immediately taken and flash-frozen. After tissue samples were homogenized, RNA was isolated and gene expression was measured using the qRT-PCR methods described above, except normalizing expression to the housekeeping gene *Actb*. Gene expression was compared between HFD and control mice via a two-sample t-test, and due to the small number of mice within each group, *P*-values were calculated using a Monte-Carlo permutation approach. A one-sided *P* -value ≤ 0.05 was considered statistically significant. All experiments were approved by the University of Florida Institutional Animal Care and Use Committee.

## Results

In total, 28 subjects were included in the RNA-seq analysis, as two IpcPH samples failed quality control and were excluded from all analyses. Demographics were similar among the three groups, as well as prevalence of hypertension, obstructive sleep apnea, lung disease, and hemodialysis use. As anticipated based on study selection criteria and diagnostic differences between PH groups, mean PVR, MPAP, TPG, and DPG were significantly different between patient groups (Table 1). Smoking status also significantly differed among all groups, since the majority of PH patients were previous smokers and roughly a third of patients without PH were current smokers.

**Table 1:** Baseline characteristics of the discovery cohort.

Baseline Characteristics	HFpEF without PH (n = 10)	IpcPH (n = 8)	CpcPH (n = 10)	*P*-value
Age (years), mean ± SD	58 ± 6	63 ± 12	55 ± 8	0.16
Female sex, n (%)	8 (80)	5 (62.5)	5 (50)	0.38
Race/ethnicity, n (%)				0.31
African American	7 (70)	3 (37.5)	7 (70)	
Latino American	0 (0)	3 (37.5)	1 (10)	
European American	2 (20)	2 (25)	2 (20)	
Unknown	1 (10)	0 (0)	0 (0)	
mPAP (mmHg), mean ± SD	N/A	33.6 ± 6.4	49.7 ± 7.5	0.001
PCWP (mmHg), mean ± SD		25.0 ± 8.5	22.5 ± 3.3	0.89
PVR (Wood units), mean ± SD		1.2 ± 0.7	4.1 ± 1.4	0.002
TPG (mmHg), mean ± SD		8.8 ± 4.1	27.2 ± 5.7	0.0004
DPG (mmHg), mean ± SD		0.5 ± 1.4	11.5 ± 3.3	0.0003
LVEF (%), mean ± SD	58.5 ± 5.7	65.0 ± 4.8	61.0 ± 5.8	0.07
Systemic hypertension, n (%)	9 (90)	8 (100)	10 (100)	1.00
Obstructive Sleep Apnea, n (%)	4 (40)	3 (37.5)	5 (50)	0.90
History of other lung disease*, n (%)	6 (60)	1 (12.5)	6 (60)	0.08
Hemodialysis, n (%)	1 (10)	1 (12.5)	2 (20)	1.00
Smoker				0.01
Never	5 (50)	3 (37.5)	1 (10)	
Previous	2 (20)	5 (62.5)	9 (90)	
Current	3 (30)	0 (0)	0 (0)	

DPG- diastolic pulmonary gradient; LVEF- left ventricular ejection fraction; mPAP- mean pulmonary artery pressure; OSA- obstructive sleep apnea; PCWP- pulmonary capillary wedge pressure; PVR- pulmonary vascular resistance; SD- standard deviation; TPG- transpulmonary gradient. *Includes asthma, chronic obstructive pulmonary disease, restrictive lung disease, latent tuberculosis, parenchymal lung disease, history of pulmonary embolism.

### Differential gene expression analysis

**Figure 1:**
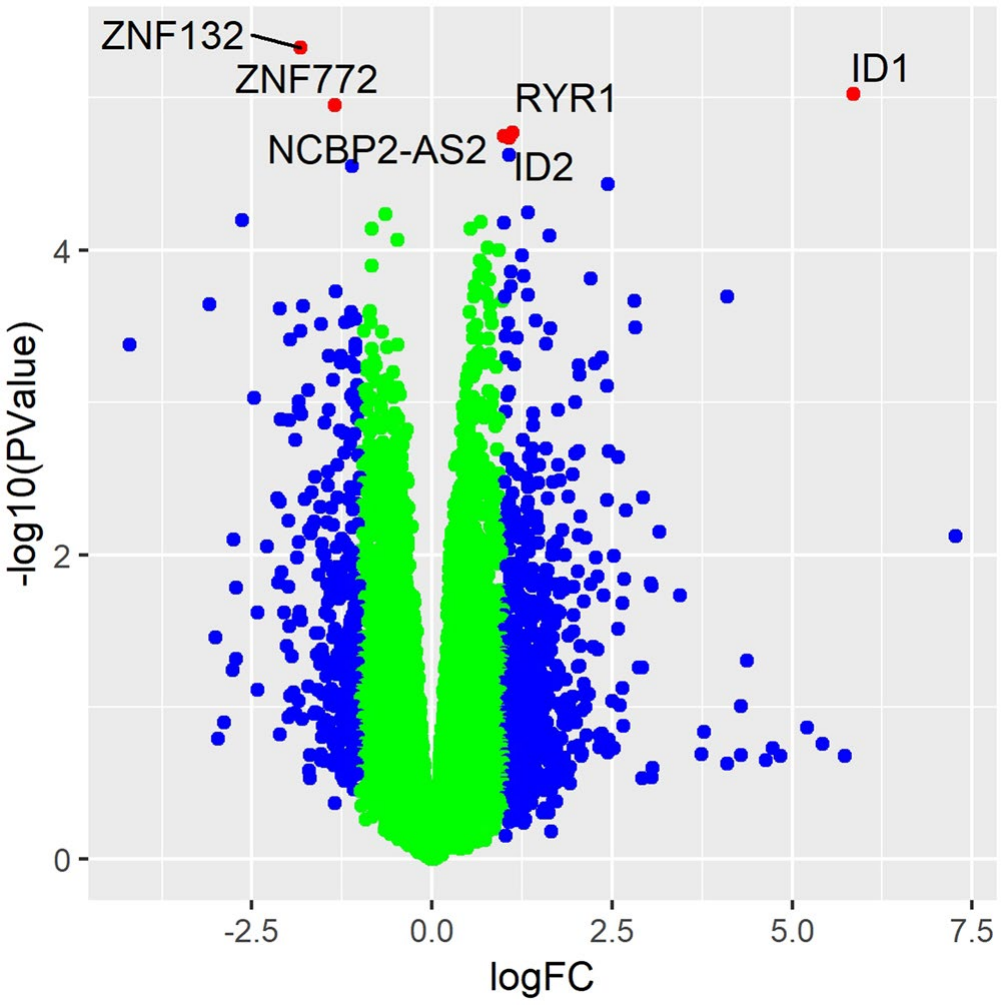
Volcano plot comparing gene expression between HFpEF subjects with CpcPH and those without PH. Blue dots represent genes with |log fold-change| > 1. Red dots represent genes with |log fold-change| > 1 and FDR < 0.05.

An average of 49.6–68.0 million paired-end reads per sample were mapped to the human reference genome (hg38), of which approximately 85% were uniquely mapped. Additional information on alignment statistics and normalization are found in the Online Data Supplement. In the adjusted analysis, four genes were significantly upregulated (*ID1*, *ID2*, *RYR1*, *NCBP2-AS2*), and two were significantly downregulated (*ZNF772*, *ZNF132*), in CpcPH patients compared to HFpEF patients without PH (FDR ≤ 0.05; Fig. 1 and Table 2). No differentially expressed genes meeting the pre-defined significance threshold were observed when comparing the no PH to IpcPH or CpcPH to IpcPH groups.

**Table 2:** Differentially expressed genes (FDR < 0.05) between HFpEF subjects with CpcPH and those without PH.

Gene Symbol	Gene Name	Log Fold-Change	Fold-Change	*P*-value
*ZNF132*	zinc finger protein 132	−1.83	0.3	4.68 × 10^−06^*
*ID1*	inhibitor of DNA binding 1	5.85	57.7	9.47 × 10^−06^*
*ZNF772*	zinc finger protein 772	−1.34	0.4	1.12 × 10^−05^*
*RYR1*	ryanodine receptor 1	1.11	2.2	1.69 × 10^−05^*
*NCBP2-AS2*	NCBP2 antisense RNA 2	1.00	2.0	1.80 × 10^−05^*
*ID2*	inhibitor of DNA binding 2	1.08	2.1	1.83 × 10^−05^*

*FDR ≤ 0.05.

### Pathway and network analyses

**Figure 2:**
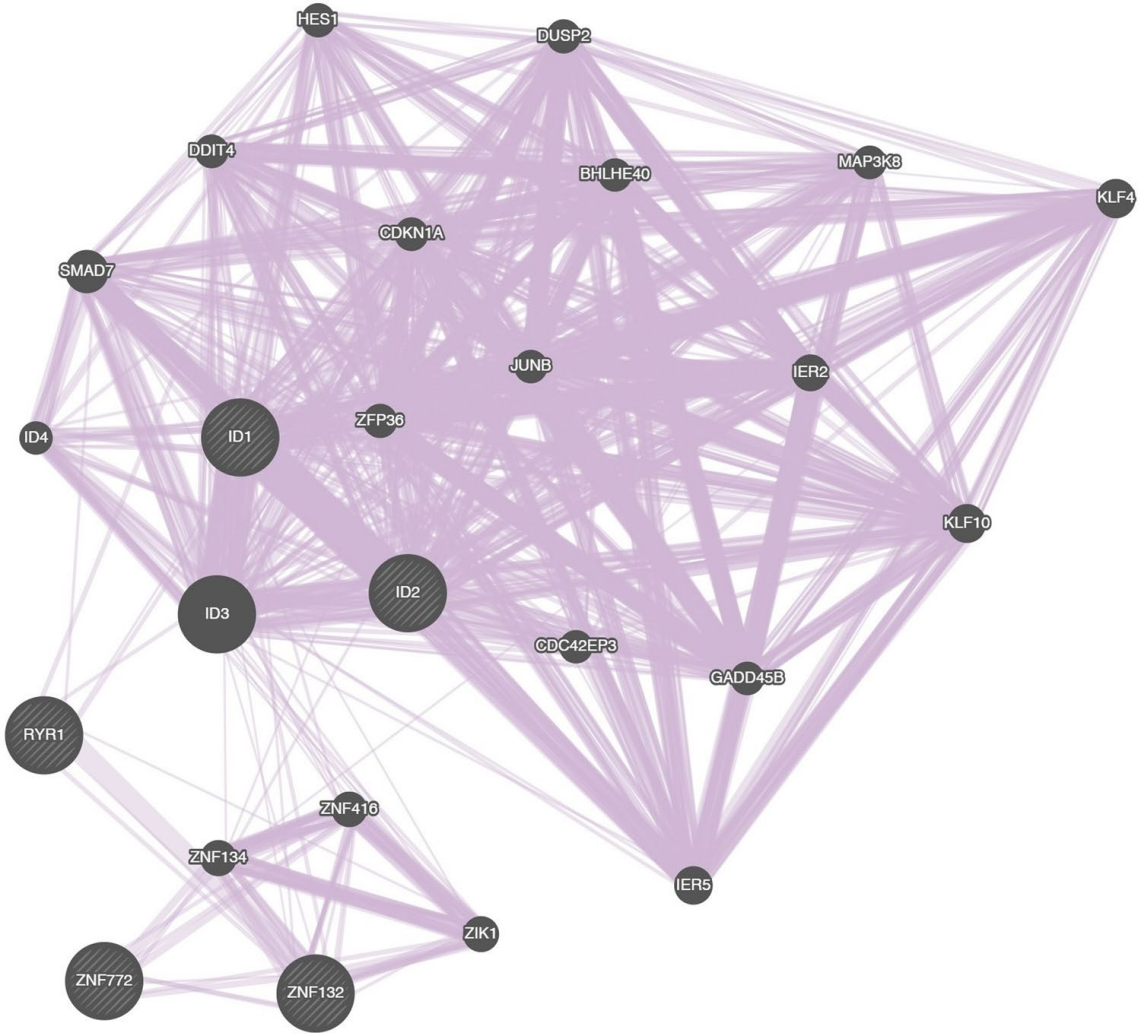
Network analysis completed for genes with significant differential expression identified in RNA-seq analysis (shaded with gray diagonal lines).

In an attempt to identify additional genes that might be involved in CpcPH pathway known to interact with genes differentially expressed CpcPH, we conducted a network analysis. A single cluster was created with 5 of the 6 genes (Fig. 2), as *NCBP2-AS2* did not cluster with any other genes. Within the network of co-expressed genes, a total of 19 additional genes were identified as part of the network and also detected in our RNA-seq analysis. Of these, over half (11 genes) were also differentially expressed in CpcPH compared to no PH with marginal associations (*P* < 0.05), and three of the 11 differentially expressed genes (*DUSP2*, *MAP3K8*, *IER5*) had associations with FDR < 0.1.

Based on enrichment analysis of all 21,680 genes detected, the “cell cycle” and “oxidative phosphorylation” KEGG pathways were significantly upregulated in CpcPH compared to subjects without PH, with nine other pathways marginally upregulated (Table 3). Similar to individual gene results, no significant pathway associations were observed when comparing IpcPH to CpcPH or IpcPH to CpcPH.

**Table 3:** KEGG pathways upregulated in CpcPH compared to HFpEF subjects without PH.

Pathway (Homo sapiens)	*P*-value
Cell cycle (hsa04110)	0.0001*
Oxidative phosphorylation (hsa00190)	0.0003*
Phagosome (hsa04145)	0.0036
Oocyte meiosis (hsa04114)	0.0044
Gap junction (hsa04540)	0.0069
p53 signaling pathway (hsa04115)	0.0071
Proteasome (hsa03050)	0.0100
Protein processing in endoplasmic reticulum (hsa04141)	0.0171
Ribosome (hsa03010)	0.0180
Regulation of actin cytoskeleton (hsa04810)	0.0323
Progesterone-mediated oocyte maturation (hsa04914)	0.0387

*FDR ≤ 0.05.

### Replication of gene expression associations

We attempted to replicate associations identified by RNA-seq within the *ID1*, *ID2*, *RYR1, ZNF772*, and *ZNF132* genes in 34 additional subjects with HF (Table 4). Overall, clinical characteristics were similar between the discovery cohort used for the RNA-seq analysis and the replication cohort, with the exception of a lower LVEF in the replication cohort (likely due to the inclusion of HFrEF patients). Out of the five genes tested for replication, only *ID2* expression was significantly upregulated in CpcPH patients compared to HF patients without PH (Table 5). Similar to the original transcriptomic analysis, none of the genes tested were associated with IpcPH (when compared to no PH or CpcPH).

**Table 4:** Demographic and clinical characteristics of the replication cohort.

Baseline Characteristics	HF without PH (n = 14)	IpcPH (n = 11)	CpcPH (n = 9)	*P*-value
Age (years), mean ± SD	55 ± 11	55 ± 13	56 ± 9	1.0
Female sex, n (%)	10 (71.4)	7 (63.6)	6 (66.7)	1.0
Race/ethnicity, n (%)				
African American	12 (85.7)	10 (90.9)	8 (88.9)	
Latino	2 (14.3)	1 (9.1)	1 (11.1)	1.0
European American	0 (0)	0 (0)	0 (0)	
mPAP (mmHg), mean ± SD	N/A	32.2 ± 5.2	37.8 ± 8.5	0.13
PCWP (mmHg), mean ± SD		27.5 ± 8.5	20.1 ± 9.0	0.08
PVR (Wood units), mean ± SD		1.7 ± 0.7	5.2 ± 2.0	0.0008
TPG (mmHg), mean ± SD		5.1 ± 3.0	17.7 ± 3.3	0.0002
DPG (mmHg), mean ± SD		0.1 ± 0.3	8.6 ± 4.3	<0.0001
LVEF (%). mean ± SD	40.4 ± 12.7	38.9 ± 9.2	40.0	0.94
Systemic hypertension, n (%)	13 (92.9)	9 (81.8)	9 (100)	0.46
Obstructive Sleep Apnea, n (%)	1 (7.1)	5 (45.5)	4 (44.4)	0.05
History of other lung disease*, n (%)	6 (42.9)	3 (27.3)	3 (33.3)	0.90
Hemodialysis, n (%)	0 (0)	0 (0)	0 (0)	N/A
Smoker, n (%)				
Never	5 (35.7)	4 (36.4)	4 (44.4)	
Previous	5 (35.7)	4 (36.4)	4 (44.4)	0.91
Current	4 (28.6)	3 (27.3)	1 (11.1)	

DPG- diastolic pulmonary gradient; LVEF- left ventricular ejection fraction; mPAP- mean pulmonary artery pressure; OSA- obstructive sleep apnea; PCWP- pulmonary capillary wedge pressure; PVR- pulmonary vascular resistance; SD- standard deviation; TPG- transpulmonary gradient. *Includes asthma and chronic obstructive pulmonary disease.

**Table 5:** Replication of six differentially expressed genes comparing HF subjects with CpcPH to those without PH.

Gene	Fold change (CpcPH vs. no PH)	*P*-value (one-sided)
*ZNF132*	0.89	0.410
*ID1*	0.81	0.546
*ZNF772*	0.75	0.156
*RYR1*	1.29	0.329
*NCBP2-AS2*	0.94	0.619
*ID2*	1.24	**0.047**

### Tissue-Specific validation of gene expression associations

**Figure 3:**
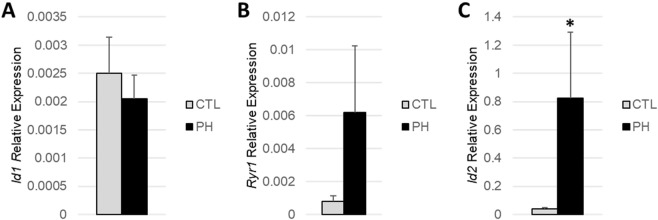
Comparison of (**A**) *Id1*, (**B**) *Ryr1*, and (**C**) *Id2*, pulmonary gene expression in HFpEF-PH mice vs. control mice. N = 3–5 in each group. *P ≤ 0.05 compared to control mice.

To confirm that gene expression signatures discovered in PBMCs were consistent in lung tissue, expression was measured in the lungs using a mouse-model of HFpEF-PH. Compared to control mice, HFpEF-PH mice had numerically higher left ventricular end diastolic pressure (LVEDP) and significantly higher right ventricular systolic pressure (RVSP), body weight, and hemoglobin A1c (Fig. S3). Because murine homologs of *NCBP2-AS2, ZNF772*, and *ZNF132* could not be identified, only *Id1*, *Id2*, and *Ryr1* were tested. Out of the three genes tested, only *Id2* was significantly upregulated in HFpEF-PH mice versus control mice (Fig. 3).

## Discussion

We conducted a transcriptome-wide analysis followed by replication and validation studies to identify genes and biological pathways that may be involved in the development of PH in HFpEF patients. To our knowledge, this is the first such study to investigate transcriptomic differences in HF-PH. Using total RNA extracted from PBMCs, we used RNA-seq to compare pairwise gene expression values among three groups: CpcPH vs. HFpEF without PH, CpcPH vs. IpcPH, and IpcPH vs. HFpEF without PH. Of these, differentially expressed genes were only discovered between CpcPH vs. HFpEF subjects without PH. Six genes were identified, including two from the “inhibitor of DNA binding” (ID) family (*ID1* and *ID2*). From a network analysis of previously established co-expressed genes, we found that 11 of 19 genes also had at least marginal associations with CpcPH, suggesting that our transcriptomic associations are not likely due to chance. In the gene set enrichment analysis, pathways involved in “cell cycle” and “oxidative phosphorylation” were significantly upregulated in CpcPH compared to subjects without PH. Following the transcriptome-wide analysis, we replicated the observed association between increased *ID2* expression and CpcPH in a separate, similar-sized cohort of both HFpEF and HFrEF patients. Finally, we completed a tissue-specific validation showing *Id2* is also upregulated in the lungs of HFpEF-PH mice.

Our results suggest that ID signaling may be involved in the development of CpcPH. The ID family consists of four members (ID1 − ID4) that are part of the helix-loop-helix (HLH) group of transcription factors. ID proteins bind with other HLH transcription factors to form heterodimers, preventing the complex binding to their specific binding motif. Previous evidence shows that ID1 - ID3 are important in the cardiovascular system, have functions closely linked to vascular endothelial growth factor (VEGF), and are associated with muscle cell differentiation as well as endothelial cell activation, differentiation, and proliferation^33^. ID2 in particular has been associated with promotion of cell death, prevention of differentiation, regulating myogenesis, vascular smooth muscle cell phenotype, and vessel plasticity^34–37^. Moreover, ID1 - ID3 are all expressed in endothelial and vascular smooth muscle cells derived from pulmonary arteries and microvasculature^38,39^. *ID* expression is regulated by transforming growth factor beta (TGFβ)-related growth factors, such as the bone morphogenetic proteins (BMPs)^40^.

BMP signaling is fairly well-described in the pathogenesis of pulmonary arterial hypertension (PAH), primarily through clinical data associating BMP receptor type II (*BMPR2*) gene mutations with familial PAH. *BMPR2* mutations can also cause pulmonary artery smooth muscle cell (PASMC) proliferation^41^. The downstream effect of BMP signaling on PASMC and endothelial cell proliferation appears to be mediated by ID proteins^42,43^. However, the exact relationship between BMP signaling, ID expression, and PH pathogenesis is not well-understood. While multiple groups have shown correlations between BMP stimulation and ID1 and ID3 upregulation (and its inverse), the response of ID2 is less clear^39,44^. In fact, ID2 appears to be more likely to be upregulated in hypoxia (a well-established stimulus of PH), and has been associated with upregulation of genes involved in smooth muscle cell proliferation^45,46^. Thus, our findings of increased *ID2* expression in patients with a PH phenotype seem to be consistent with previous findings.

To our knowledge, we are the first to report that *ID* gene expression is associated with CpcPH development. While the exact mechanism is unclear, the association of ID proteins with BMP downstream signaling and PAH development is reasonably well-established. Thus, our finding of differential ID2 expression in CpcPH patients suggest that alterations in the BMP signaling pathway (via at least ID2) could play a role in development of CpcPH, perhaps in a similar fashion as in PAH. This theory is supported by the work of Assad *et al*., who have demonstrated that CpcPH patients have a pulmonary vascular physiology that more closely resembles that of PAH patients than that of IpcPH patients^47^. However, additional research is needed to better outline the mechanisms underlying ID function in CpcPH.

Evidence suggests that PH is a multifaceted pan-vasculopathy that mirrors characteristics observed in cancer, including resistance to apoptosis, inflammation, increased proliferation, and fibrosis caused by remodeling of the extracellular matrix^48^. Dysregulation in the ability of pulmonary vascular cells to progress through the cell cycle stages could result in apoptosis resistance and cell proliferation. Thus, it is not surprising that our pathway analysis identified significant upregulation of the “cell cycle” pathway in CpcPH patients. In fact, ID proteins have been associated with promotion of proliferation and migration of endothelial progenitor cells, and markers of smooth muscle cell proliferation^45,49^.

A popular theory contends that early in PH development, initial endothelial cell apoptosis causes a separate population of pathogenic and hyperproliferative endothelial cells that drive later PH pathogenesis^50–52^. In a phenomenon discovered in cancer called the Warburg effect, cells rely upon glycolysis and shift away from oxidative phosphorylation for production of cellular energy^53^. However, aerobic glycolysis is not limited to malignancy, as the Warburg effect has also been described to play a role in PAH pathogenesis^54–56^. Interestingly, evidence shows that ID2 regulates mitochondrial function and contributes to maintenance of mitochondrial membrane potential, oxidative respiration, and mitochondrial electron transport chain functions^57^. Specifically, *ID2* has shown the ability to suppress mitochondrial oxidative respiration and ATP production^57^. However, our pathway analysis showed that the “oxidative phosphorylation” pathway was significantly upregulated in CpcPH patients compared to those without PH. Thus, our findings seem to contradict this theory and contrast previous evidence showing a repression of endothelial oxidative phosphorylation in PH development^58^. This apparent discrepancy could simply reflect differences in gene expression patterns between the pulmonary vasculature and circulating immune cells. Further research is needed to clarify these differences.

Another gene identified in the RNA-seq analysis, *RYR1* (which encodes the rynodyne receptor 1), was consistently upregulated in each analysis. *RYR1* is primarily expressed in skeletal muscle, but is also expressed to a lesser extent in vascular smooth muscle. Its role in calcium-dependent muscle contraction makes it a potential candidate gene for PH development. However, neither the replication nor validation analysis showed significant associations and network analysis did not identify any additional genes with even nominal associations between CpcPH and subjects without PH. Thus, we did not find sufficient evidence associating *RYR1* with CpcPH pathogenesis.

While we identified multiple differentially expressed genes in our transcriptome-wide analysis, we found no associations with any comparisons including IpcPH subjects. This lack of significant associations could suggest that those analyses were underpowered. This could be due to either the loss of two IpcPH samples that were excluded because of a failure to meet quality control standards. Or, as discussed above, perhaps a much larger sample size is needed to detect differentially expressed genes when comparing IpcPH to subjects without PH because those phenotypes appear more related to each other than to CpcPH.

This study has several strengths and limitations. First, we identified through transcriptome-wide analysis that *ID2* expression is significantly increased in the blood of CpcPH patients compared to those without PH and translated these results to show similar expression in the lung. An additional strength of this study is the quality of our phenotype data collected on patients, as we had extensive echocardiogram, clinical history, and medication data, including right heart catheterization data on the patients with PH. Accompanied by the high throughput sequencing data, this detailed phenotype data likely contributed to our ability to identify differentially expressed genes between CpcPH and no PH, in spite of a relatively small sample size.

Our relatively small sample size in each PH group could also be a limitation, potentially underpowering us to detect additional gene expression associations. Because of the difficulty obtaining lung biopsies from PH patients, we opted to complete our transcriptome-wide analysis in PBMCs, which is not likely the primary tissue involved in PH development. In order to consider ID2 expression levels in PBMCs to be a reliable predictor of CpcPH development, additional studies would be required showing that changes in ID2 expression are associated with corresponding alterations in PH phenotype. Our data from a murine model of HFpEF-PH indicate that *ID2* is similarly upregulated in the lungs. However, total lung tissue was used in our validation rather than isolated pulmonary vascular tissue which may have also decreased sensitivity for validating additional gene expression associations. Additional mechanistic studies are needed to better parse out the cell-specific mechanism of ID signaling in CpcPH. Lastly, we are unable to rule out that some of the subjects classified as CpcPH were actually PAH subjects with left HF, since there is currently no practical way to clinically differentiate between these conditions^59^. This highlights the need to reclassify PH based on molecular phenotype^60^ due to challenges presented by the current classification system, which have ultimately impacted our ability to identify innovative treatment strategies for patients with Group 2 PH^61^. Further research into the role of the ID signaling pathway in CpcPH development could be one way to contribute towards this unmet need.

## Supplementary information

Supplementary Data

## Data Availability

The datasets used and/or analysed during the current study are available from the corresponding author on reasonable request.
