# Supplementary material for: Transcriptome-wide analysis associates ID2 expression with combined pre- and post-capillary pulmonary hypertension
Source: Sci Rep. 2019 Dec 20;9:19572. doi: 10.1038/s41598-019-55700-y (PMC6925238; doi:10.1038/s41598-019-55700-y)
Supplement: Supplementary file 1 — Supplementary Data [file 41598_2019_55700_MOESM1_ESM.docx]

**SUPPLEMENTAL MATERIAL**

**Transcriptome-wide analysis associates *ID2* expression with combined pre- and post-capillary pulmonary hypertension**

Meghan J. Arwood^1^*****, Nasim Vahabi^1^*****, Christelle Lteif^1^, Ravindra K. Sharma^2^, Roberto F. Machado^3^, and Julio D. Duarte^1^******

1. Department of Pharmacotherapy and Translational Research, College of Pharmacy, University of Florida, Gainesville, FL, USA
2. Department of Physiology and Functional Genomics, College of Medicine, University of Florida, Gainesville, FL, USA
3. Department of Medicine, Division of Pulmonary, Critical Care, Sleep, and Occupational Medicine, Indiana University, Indianapolis, IN, USA

*****These authors contributed equally

**** Corresponding author:** Julio D. Duarte; 1600 SW Archer Road, Gainesville, FL 32610-0486, USA; Email: [juliod@cop.ufl.edu](mailto:juliod@cop.ufl.edu); Telephone: 352-273-8132

**SUPPLEMENTAL METHODS**

**RNA-seq Analysis:** Before beginning differential expression analysis, genes/transcripts with low expression were filtered out, keeping genes/transcripts with a count per million (CPM) value greater than 0.2. Since the minimum library size (sequence-depth) was 21,790,929 reads, genes/transcripts were kept in the analysis if they had five fragments in at least five samples (${cutoff}_{CPM}=\frac{5}{Min (Library-size)}\times1,000,000$). After filtering, the number of genes were 21,680 which were used for differentially gene expression analysis.

The trimmed mean of M-values (TMM) method(1) was used to normalize RNA-seq data for composition bias and to estimate the relative RNA expression. TMM normalization was performed using the *calcNormFactor* function, which calculated a normalization factor for each sample. The product of these factors and the library sizes defined the effective library size in all downstream analyses.(2) In order to explore the differences between libraries as a quality control check, mean-difference plots were generated for all samples. Each plot compared the expression log-ratio between the corresponding sample and the average log-expression across all the other libraries.

Since the final data for differential gene/transcript expression and downstream analyses were “count”, most of them exhibited over/under-dispersion. For this reason, the negative-binomial regression model is widely used and has been reasonably successful in modeling bulk RNA expression.(3) Moreover, using a quasi-likelihood method enabled us to take into account the uncertainty in variance and overdispersion within the dataset by defining an overdispersion parameter (using the *estimateDisp* function in edgeR). The *estimateDisp* function estimates both the empirical Bayes moderated dispersion (for each gene) and the common dispersion (global dispersion estimate averaged over all genes).

In order to examine which molecular pathways were most enriched based on the differential expression results, we conducted a gene set enrichment analysis using a functional class scoring method called Generally Applicable Gene set Enrichment (GAGE).(4) Functional class scoring methods address the limitations of over-representation analysis approaches. They do not require an arbitrary threshold for gene significance and use all available gene expression measurements to detect the coordinated changes in gene expression to identify pathways containing significant gene expression changes.(5) We used pathways derived from Kyoto Encyclopedia of Genes and Genomes (KEGG).(6) The pathway analysis was performed using the *gage* function in R.

**SUPPLEMENTAL RESULTS**

**RNA-seq alignment and normalization**

An average of 49.6–68.0 million reads per sample were mapped to the human reference genome (hg38). The alignment statistics are presented in Table E1. The mean (SD) rate of uniquely-mapped, mismatched (per base) and unmapped reads were 85.86% (3.66%), 0.23% (0.02%) and 0.07% (0.02%), respectively, which suggests successful sequence alignment.

The density plots of the log-CPM values before and after filtering are shown in Figure E1. A large proportion of genes had low expression before filtering, but applying the CPM threshold of 0.2 discarded a significant amount of them. After filtering, the number of genes/transcripts available for differential expression analysis was reduced from 60,604 to 21,680. Mean library sizes for each group of HFpEF samples after TMM normalization are presented in Table S2. The mean-difference plots for all samples are shown in Figure S2. In this figure, most of the genes/transcripts are centered around the line of zero expression log-ratio in all samples, which suggests there was no composition bias in our RNA-seq data.

**SUPPLEMENTAL TABLES**

**Table S1.** Alignment statistics from STAR as a post-alignment quality control tool.

| **RNA-seq read pairs*** | **No PH**  **(n=10)** | **IpcPH**  **(n=8)** | **CpcPH**  **(n=10)** | **Total**  **(n=28)** |
| --- | --- | --- | --- | --- |
| **Total number of reads** | 581,839,436 | 451,993,807 | 556,558,150 | 1,590,391,393 |
| **Average number of reads/sample** | 58,183,944 (5,746,033.9) | 56,499,226 (6,378,208.4) | 55,655,815 (6,208,033.0) | 56,799,693 (5,968,275.2) |
| **Average input length** | 150.1 | 150 | 150.2 | 150.1 |
| **Alignment %** | 99.84 (0.03) | 99.83 (0.02) | 99.83 (0.04) | 99.83 (0.03) |
| **Uniquely mapped %†** | 85.72 (2.53) | 86.94 (2.94) | 85.14 (5.04) | 85.91 (3.54) |
| **Multi-mapped %†** | 14.12 (2.56) | 12.88 (2.94) | 14.69 (5.06) | 13.89 (3.52) |
| **Un-mapped %** | 0.07 (0.02) | 0.07 (0.02) | 0.06 (0.02) | 0.07 (0.02) |
| **Mismatched %**‡ | 0.22 (0.02) | 0.23 (0.02) | 0.24 (0.02) | 0.23 (0.02) |
| * Reported as mean (SD), unless otherwise noted  **†** Ratio of Uniquely/Multi mapped = 6.13% (acceptable range for humans is <20%)  ‡ Acceptable range is <0.80% (shows the library quality) | | | | |

**Table S2.** Descriptive statistics for library sizes after TMM normalization on RNA-seq read-counts.

| **Statistic** | **Group** | | | **Overall** |
| --- | --- | --- | --- | --- |
|  | **No PH** | **IpcPH** | **CpcPH** |  |
| **Mean** | 32,178,981 | 30,644,283 | 29,916,024 | 30,932,297 |
| **Min** | 24,803,334 | 28,617,513 | 21,790,929 | 21,790,929 |
| **Max** | 38,193,070 | 33,950,414 | 39,440,262 | 39,440,262 |

**SUPPLEMENTAL FIGURES**

**
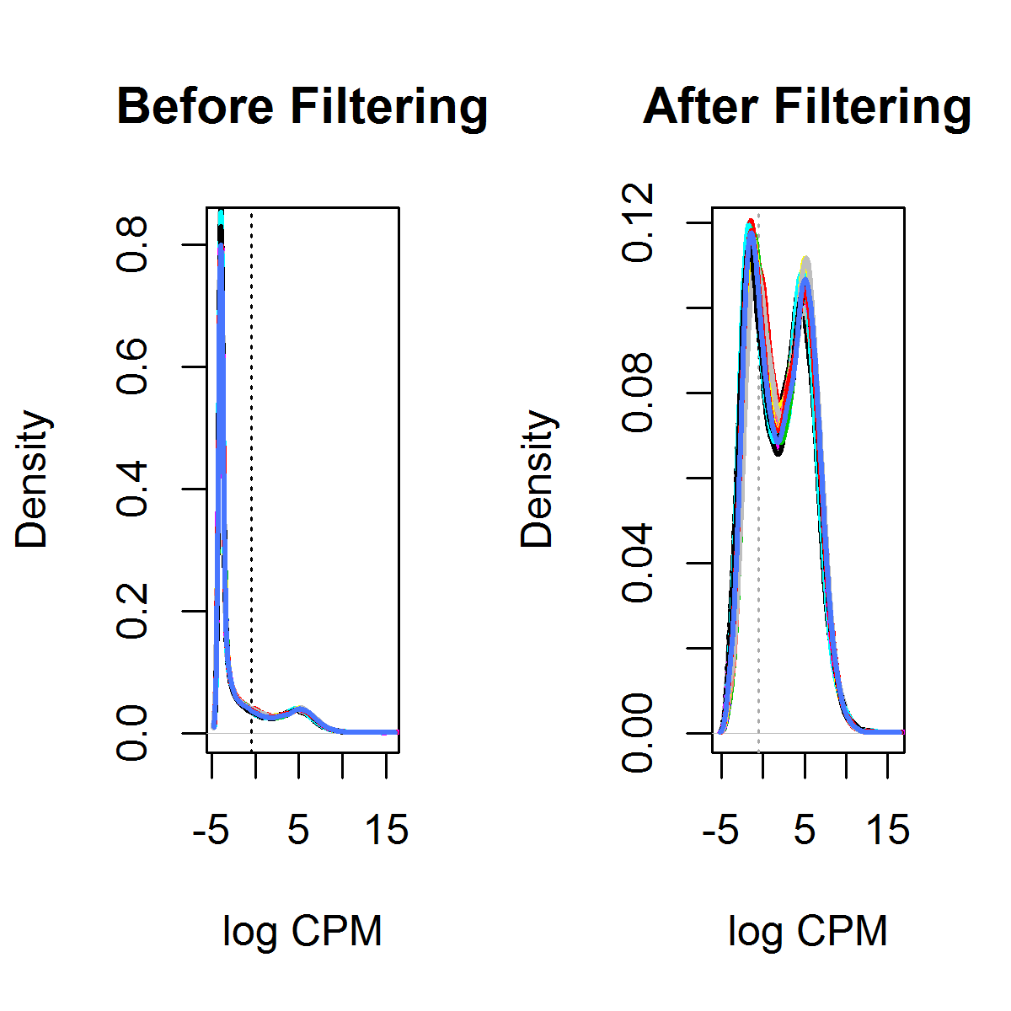
**

**Figure S1.** The density of log-CPM values before and after filtering the lowly expressed genes. Dotted vertical lines define the log-CPM of -0.5 threshold, which is equivalent to a CPM value of 0.2, used in the filtering step as the expression cutoff.

**
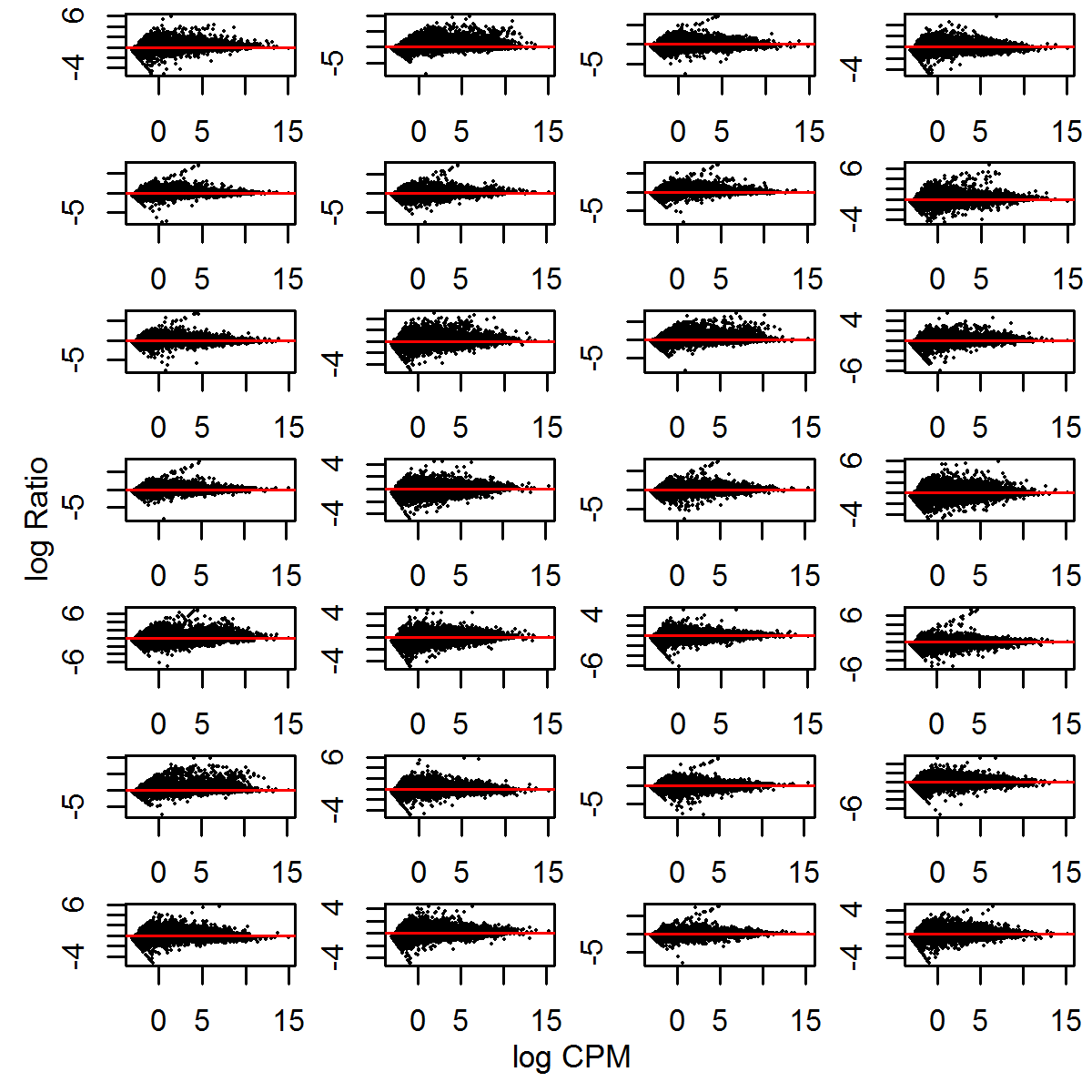
**

**Figure S2.** Mean-difference plot for all HFpEF patients. Each point represents a gene, and the red line indicates a log-ratio of zero.

**
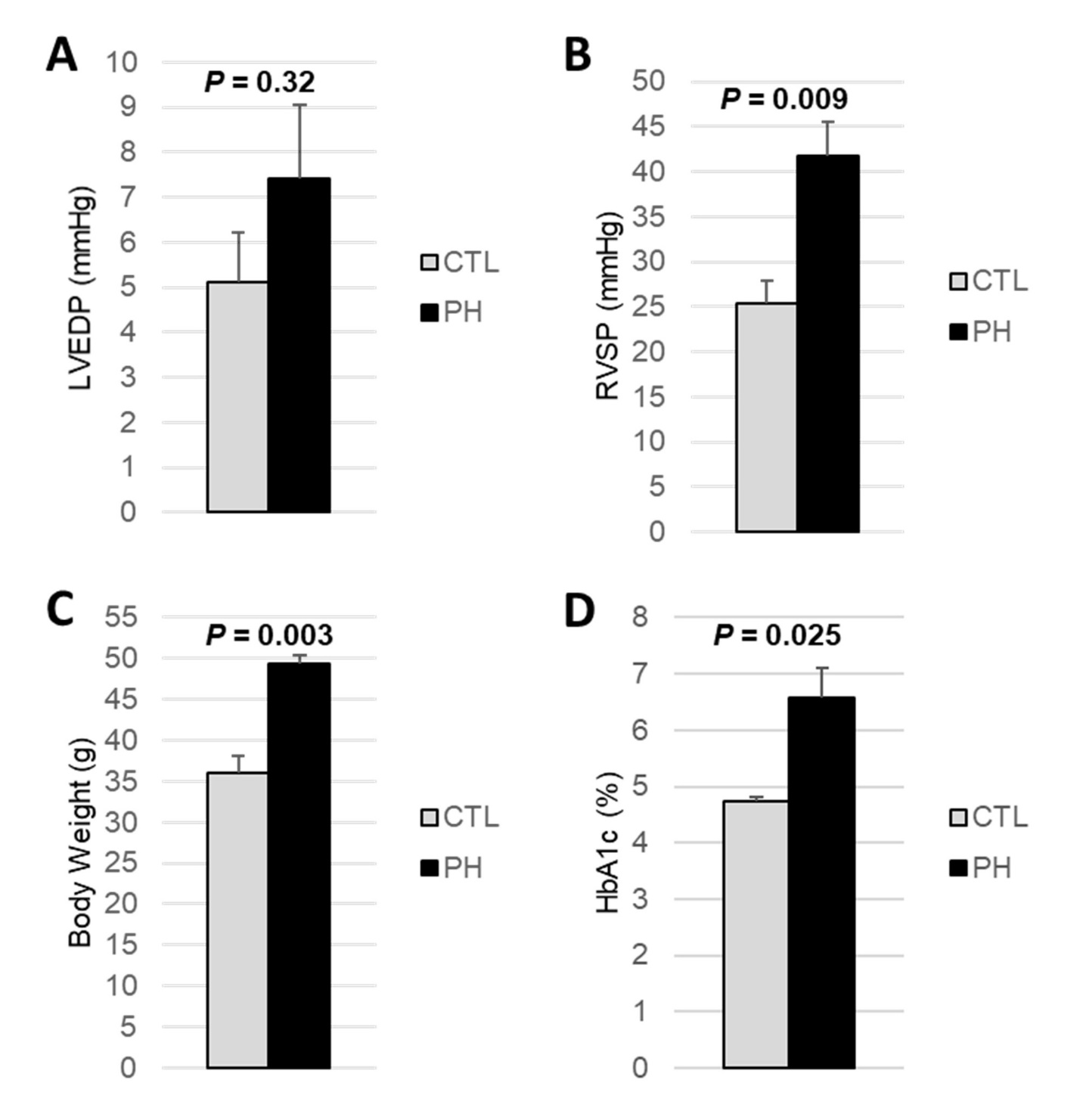
**

**Figure S3.** Comparisons of (A) LVEDP, (B) RVSP, (C) body weight, and (D) HbA1c between HFpEF-PH and control mice. N = 4-5 in each group.

**SUPPLEMENTAL REFERENCES**

1. Robinson MD, Oshlack A. A scaling normalization method for differential expression analysis of RNA-seq data. *Genome Biol* 2010; 11: R25.

2. Chen Y, Lun AT, Smyth GKJF. From reads to genes to pathways: differential expression analysis of RNA-Seq experiments using Rsubread and the edgeR quasi-likelihood pipeline. 2016; 5.

3. Datta S, Nettleton D. Statistical analysis of next generation sequencing data. Springer; 2014.

4. Luo W, Friedman MS, Shedden K, Hankenson KD, Woolf PJ. GAGE: generally applicable gene set enrichment for pathway analysis. *BMC Bioinformatics* 2009; 10: 161.

5. Khatri P, Sirota M, Butte AJ. Ten years of pathway analysis: current approaches and outstanding challenges. *PLoS Comput Biol* 2012; 8: e1002375.

6. Kanehisa M, Goto SJNar. KEGG: kyoto encyclopedia of genes and genomes. 2000; 28: 27-30.
